# Possible Misidentification of *Mycobacterium yongonense*

**DOI:** 10.3201/eid2006.131508

**Published:** 2014-06

**Authors:** Sung Kuk Hong, Eui-Chong Kim

**Affiliations:** Seoul National University Hospital, Seoul, South Korea

**Keywords:** Mycobacterium yongonense, Mycobacterium marseillense, Mycobacterium intracellulare, tuberculosis and other mycobacteria, bacteria, identification, 16S rRNA, hsp65, rpoB, sodA, gene, internal transcribed spacer 1

**To the Editor:** Tortoli et al. (*1*) reported pulmonary disease caused by *M. yongonense* strains isolated from patients in Italy; these strains were identified by sequencing the 16S rRNA, *hsp65*, *rpoB*, and *sodA* genes and the internal transcribed spacer 1 (ITS1) region. The 16S rRNA gene sequence of these isolates showed 100% similarity with those of *M. yongonense* and *M. marseillense*. The isolates were more closely related to *M. yongonense* than to *M. marseillense* in terms of the *hsp65* gene and ITS1 region; however, the *rpoB* gene sequence showed a higher degree of similarity to that of *M. intracellulare* (99.4%) than to that of *M. marseillense* (97.4%). The authors did not mention the similarity of the isolates with *M. intracellulare* in these sequences except for the *rpoB* gene. However, because these sequences showed high similarity to *M. yongonense*, a high degree of similarity to *M. intracellulare* could be inferred.

The initial description of *M. yongonense* highlighted its unique molecular character (*2*). The 16S rRNA and *hsp65* genes and ITS1 region are closely related to those of *M. intracellulare* ATCC 13950^T^; however, the *rpoB* gene is closely related to that of *M. parascrofulaceum* ATCC BAA-614^T^ (99.4%). No consensus guidelines are available for mycobacterial identification, but the *rpoB* gene has been used widely as a target gene; multilocus sequence analysis also has been used recently (*3*,*4*). Although the authors suggest that a variant of *M. yongonense* preceded the acquisition of the *rpoB* gene from *M. parascrofulaceum* by a lateral gene transfer event (*3*), the isolates described are more similar to *M. intracellulare* than to *M. yongonense* on the basis of the *rpoB* gene sequence and multilocus sequence analysis. It is also possible that the isolates are a *M. yongonense* strain that preceded the acquisition of the *rpoB* gene but that are not the same as the initially described *M. yongonense*.
